# The 980 nm diode laser treatment for non-muscle-invasive bladder tumor with en bloc technique: single-center experience

**DOI:** 10.1186/s12957-022-02786-w

**Published:** 2022-09-29

**Authors:** Tianci Mao, Hongyi Zhang, Jie Cui, Zhiguang Zhao, Dian Jiao, Wei Zhang

**Affiliations:** 1 Department of Urology, Tangdu Hospital, Air Force Medical University, Xi’an, Shaanxi China; 2 Department of Urology, The First Affiliated Hospital, Xi’an Medical University, Xi’an, Shaanxi China; 3 Department of Oncology, The First Affiliated Hospital, Xi’an Medical University, Xi’an, Shaanxi China

**Keywords:** Bladder cancer, 980 nm diode laser, En bloc resection

## Abstract

**Background:**

Transurethral resection of the bladder tumor (TURBT) is one of the most established urological procedures for the treatment of the primary non-muscle-invasive bladder cancer (NMIBC). The aim of the study is to evaluate the efficacy and safety of 980 nm diode laser as a treatment for primary NMIBC.

**Methods:**

Eighty-eight patients with NMIBC were treated by en bloc transurethral resection with 980 nm diode laser, and 76 patients were treated by plasmakinetic transurethral resection from May 2016 to July 2019 at the Department of Urology, Tangdu Hospital, Air Force Medical University. The clinical data were collected and compared between the two groups.

**Results:**

The bladder irrigation time was shortened in 980 nm diode laser group compared to that of plasmakinetic transurethral resection group (4.1 ± 0.6 vs 13.1 ± 3.1 h, *p* < 0.001). A total of 13.2% (10/76) patients experienced obturator nerve reflex, and 5.3% (4/76) experienced delayed bleeding in plasmakinetic transurethral resection group, while no obturator nerve reflex and delayed bleeding cases were observed in 980 nm diode laser group (*p* < 0.05). The postoperative catheterization and hospitalization time showed no significant difference between the two groups. The median follow-up time was 27 months (13–38 months). No significant difference in the recurrence rate was observed between the two groups.

**Conclusions:**

The 980 nm diode laser is an effective and safe tool in transurethral resection of NMIBC using en bloc technique. It has less perioperative complications and shortened bladder irrigation time.

## Introduction

Bladder cancer is the most common genitourinary malignant tumor worldwide. It is more common in males than in females. In the USA, an estimated 16,000 bladder cancer-related deaths occurred in 2015. Amongst 74,000 estimated new cases of bladder cancer diagnosed in 2015, non-muscle-invasive bladder cancer (NMIBC) accounted for approximately 75% [1], and this percentage was even higher amongst younger patients (< 40 years old) [2]. NMIBC can be treated with endourological procedures.

Conventional transurethral resection of the bladder tumor (TURBT) is one of the most established urological procedures and strongly recommended by guidelines of various countries [3]. However, the numerous complications, such as bleeding, bladder perforation, and obturator nerve reflex, should still be addressed [4]. Lasers are now widely used in urologic surgery. Transurethral laser resection of bladder tumor, using holmium (Ho:YAG) or thulium (Tm:YAG), has been proved to be safe, effective, and minimally invasive for NMIBC [5]. Many studies have suggested that laser en bloc resection is superior to conventional TURBT with regard to the decreased complication rate and recurrence rate. Moreover, laser en bloc resection can provide an accurate tumor stage and grade [6, 7]. A diode laser with wavelength of 980 nm has been reported to provide effective hemostasis and vaporization of prostate [8]. However, studies on bladder tumor are limited. This study aimed to evaluate the efficacy and safety of en bloc transurethral resection of bladder tumor with 980 nm diode laser in patients with NMIBC.

## Materials and methods

### Data collection

From May 2016 to July 2019, 164 patients who were diagnosed with NMIBC and treated by either plasmakinetic transurethral resection (PK-TURBT) or 980 nm diode laser en bloc resection at the Department of Urology, Tangdu Hospital, Air Force Medical University were retrospectively investigated. The Ethics Committee of Tangdu Hospital of Air Force Military Medical University approved these procedures (Approval Number: TDLL-201601-03). The inclusion criteria included clinical stage Ta-T1N0M0, except tumor in situ, recurrent bladder cancer, and the absence of other tumors. The pathological results were non-muscle-invasive bladder cancer, and the diameter of the tumor was less than 5 cm. Clinical assessments were made, which included detailed history, physical examination, blood routine examination, urine routine examination, serum biochemical analysis, coagulation time test, and thoracic, abdominal, pelvis CT scans, and CT urography. Cystoscopy and biopsy were performed to determine the location, tumor volume, and number of multiple tumors. The preoperative clinical stage was assessed. All patients signed an informed consent form.

**Fig. 1:**
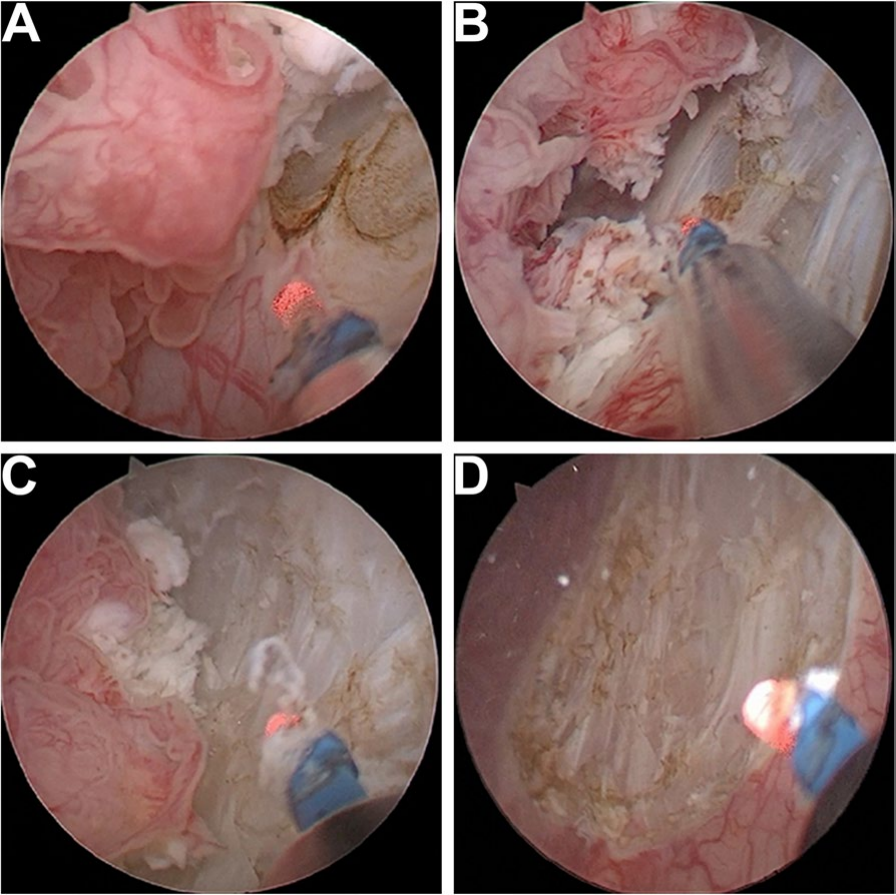
The surgery procedure of 980 nm diode laser en bloc resection of bladder tumor. **A** A circumferential incise was made about 0.5–1 cm away from the boundary of tumor. **B** The incision was deepened into the bladder muscular layer under the tumor. Then sharp and blunt dissection was performed between the muscular layer and the serosa of the bladder. **C** The incision was made along layer between the muscular layer and the serosa of the bladder. Lastly, the tissue mass was completely removed. **D** The tumor was en bloc resected, and the wound surface was observed

The standard procedures were performed in all the operations. All the surgeries were performed by one skilled surgeon in our department. All the patients were in the lithotomy position and under general or continuous epidural anesthesia. For the 980 nm diode laser group, transurethral laser en bloc resection of the bladder tumor was performed using a 24F continuous-flow cystoscope with 30° lens (Karl Storz, Germany) and 980 nm diode laser surgery system (HU120, Longhuiheng Medical Co., Ltd., China) with an average power of 120 W. Surgery was performed using a 600 μm laser fiber. The procedure is presented as follows. Firstly, the tumor location, volume, appearance, number, and adjacent mucosa were examined. Secondly, a circumferential incision about 0.5–1 cm away from the boundary of the tumor was made with a 980 nm diode laser. This step was not necessary for bulky tumors. For large tumors, we start from the accessible tumor boundary (Fig. 1A). Thirdly, the incision deepened progressively into the bladder muscular layer under the tumor. A sharp and blunt dissection was performed between the muscular layer and the serosa of the bladder using the 980 nm diode laser (Fig. 1B). Under the assistance of sheath and water stream, an operation space between the tumor base and serosa was made. At this point, the tissue mass was completely removed (Fig. 1C). Subsequently, the coagulation of the tumor base and surrounding mucosa was performed (Fig. 1D). Finally, the tissue block was rinsed out of the bladder using Ellik’s evacuator or removed by an electrocautery resection loop.

For the PK-TURBT group, a Wolf 26F continuous-flow resectoscope with plasmakinetic loop electrode (ScanMed, Zhuhai, China) were used. The cutting and coagulation power of the bipolar generator were set at 160 and 80 W, respectively. All patients underwent traditional piecemeal resection until reaching the muscular layer of the bladder. The resection margin was about 1 cm away from the tumor edge. The tumor base and surrounding mucosa were carefully coagulated, and the tumor was completely resected. The tissue was endoscopically collected by an Ellik evacuator. Pathological assessment was performed by one pathologist with the standard protocol in the Department of Pathology, Tandu Hospital, Air Force Medical University. In 980 nm diode laser group, the complete tissue specimen was stretched. Its basement and margin were stained. Then, the serial section from the resected specimen was made for pathological assessment. The traditional pathological method was performed in PK-TURBT group. Twenty-four patients in TURBT group were excluded from the study due to the lack of muscularis propria in the specimens. There was no lack of muscularis propria in 980 nm diode laser group.

Prophylactic bladder irrigation was not necessary for the patients who had clear urine after surgery. The intravesical instillation therapy with 40 mg of pirarubicin was performed in patients without gross hematuria within 24 h after surgery, and it was performed weekly for 8 weeks and then monthly for 10 months. Patients with T1 and/or high-grade bladder tumor underwent re-resection after 4 weeks. The ultrasound and cystoscopy were performed every 3 months.

### Statistical analysis

Continuous variables were summarized as mean ± SD and analyzed by Student’s *t*-test. Categorical variables were analyzed using the chi-square test or Fisher’s exact test. Statistical analysis was performed with SPSS version 19.0 software (SPSS Inc., Chicago, IL, USA). *p*-values < 0.05 were considered statistically significant.

## Results

The clinical characteristics of the patients undergoing 980 nm diode laser or PK-TURBT were listed in Table 1. There was no significant difference in the sex, age, mean tumor number and size, tumor multiplicity, location, pathological tumor stage, and grade between the two groups.

**Table 1:** Patient characteristics in 980 nm diode laser group and PK-TURBT group

Variable	980 nm diode laser (*n* = 88)	PK-TURBT (*n* = 76)	*p*-Value
Sex (*n*, %)			
Male	70 (79.5)	57 (75)	0.58
Female	18 (20.5)	19 (25.0)	
Age, mean ± SD (years)	66.0 ± 8.3	68.2 ± 6.8	0.07
Mean tumor number	1.3 ± 0.5	1.4 ± 0.6	0.36
Mean tumor size (n, %)			
≤ 3 cm	67 (76.1)	60 (78.9)	0.71
> 3 cm	21 (23.9)	16 (21.1)	
Tumor multiplicity (*n*, %)			
Single	67 (76.1)	54 (71.1)	0.48
Multiple	21 (23.9)	22 (28.9)	
Location (*n*, %)			
Lateral	57 (64.8)	50 (65.8)	1.00
Other	31 (35.2)	26 (34.2)	
Stage (*n*, %)			
Ta	46 (52.3)	33 (43.4)	0.28
T1	42 (47.7)	43 (56.6)	
Grade (WHO 2004) (*n*, %)			
PUNLMP	23 (26.1)	18 (23.7)	0.26
Low	47 (53.4)	34 (44.7)	
High	18 (20.5)	24 (31.6)	

*PUNLMP* papillary urothelial neoplasms of low malignant potential, *PK-TURBT* plasmakinetic transurethral resection of bladder tumor, *WHO* World Health Organization

Table 2 presented the intraoperative and postoperative data of the two groups. The bladder irrigation time (4.1 ± 0.6 vs 13.1 ± 3.1 h, *p* < 0.001) in the 980 nm diode laser group was shortened than that of PK-TURP group. Bladder irrigation was not performed in patients with clear urinary tubes. For patients with small amount of bladder bleeding after surgery, we performed bladder irrigation at a speed of 1000 ml/h from the beginning. When the bleeding was decreased, the bladder irrigation speed was reduced. The irrigation was stopped when the color of the urinary tube kept clear at 30 drops per minute for 1 h. None of the patients in the 980 nm diode laser group experienced obturator nerve reflex, while 10 (13.2%) patients in the PK-TURBT group experienced obturator nerve reflex during surgery. No delayed bleeding occurred in the 980 nm diode laser group. However, 4 (5.3%) patients experienced delay bleeding in the PK-TURBT group and were treated electric cauterisation or irrigation with ice physiological saline. One (1.1%) patient in the 980 nm diode laser group and 3 (3.9%) patients in PK-TURBT group experienced bladder perforation. These patients were treated with catheterization for about 30 days. After the cystography showed no bladder leakage, the catheter was removed. Forty-four (50.0%) patients in 980 nm diode laser group and 47 (61.8%) patients in PK-TURBT group were treated by secondary surgery. No tumor residuals were found in 980 nm diode laser group, while the tumor residuals were found in 2 patients in PK-TURBT group. The tumor residuals were confirmed by postoperative pathology. No patients underwent TUR syndrome, and none of the patients in the two groups needed blood transfusion. The postoperative catheterization and hospitalization time showed no significant difference between the two groups. The median follow-up time of 980 nm diode laser group and PK-TURBT group was 28 months (15–40 months) and 27 months (13–37 months), respectively. No significant difference in the recurrence rate was observed between the two groups.

**Table 2:** Intra- and postoperative characteristics in 980 nm diode laser group and PK-TURBT group

Variable	980 nm diode laser (*n* = 88)	PK-TURBT (*n* = 76)	*p*-Value
Operation time (min)	37.7 ± 4.6	38.7 ± 3.5	0.14
Obturator nerve reflex (*n*, %)	0 (0)	10 (13.2)	< 0.001
TUR syndrome	0	0	-
Bladder perforation (*n*, %)	1 (1.1)	3 (3.9)	0.34
Bladder irrigation (h)	4.1 ± 0.6	13.1 ± 3.1	< 0.001
Delayed bleeding (*n*, %)	0 (0)	4 (5.3)	0.04
Catheterization time (d)	4.3 ± 1.0	4.5 ± 1.0	0.32
Hospitalization time (d)	3.1 ± 0.3	3.2 ± 0.5	0.17
Secondary surgery (*n*, %)	44 (50.0)	47 (61.8)	0.13
Recurrence (*n*, %)	11 (12.5)	8 (10.5)	0.81

*TUR* transurethral resection, *PK-TURBT* plasmakinetic transurethral resection of bladder tumor

## Discussion

Conventional TURBT is one of the most common technique for NMIBC treatment. However, complications such as intraoperative bleeding and obturator nerve reflex often occur [4]. Moreover, the exfoliated cancer cells during operation increase the risk of recurrence and metastasis [9]. Although bipolar resection has been reported to reduce the risk of complications when compared with monopolar resection, the results remain controversial [10, 11].

To overcome the limitations of TURBT, the laser has attracted clinicians’ attention. The first use of laser in urological surgery was reported by Staehler et al. in 1978 [12]. They described the transurethral vaporization resection of urinary bladder tumors with an Nd:YAG laser. The development of the en bloc technique makes lasers be widely used for bladder tumor treatment [13]. This technique is characterized by high-quality specimens that are beneficial for pathological analysis, in which the detrusor muscle was found in 96–100% of cases. In addition, laser en bloc resection reduces the risk of scattered tumor cells during surgery [14]. The safety and efficacy of lasers including thulium laser, holmium laser, 2 μm laser, and 1.9 μm vela laser have been verified in clinical practice [15–18]. The 980 nm diode laser has been demonstrated to be suitable for NMIBC treatment in an in vitro model [19]. A study from Clemente Ramos L. M. demonstrated that prostate vaporization using the 980 nm diode laser with output power up to 120 w is effective and related to minimal morbidity [20]. To date, there were less studies about the vaporization resection of NMIBC with 980 nm diode laser.

**Fig. 2:**
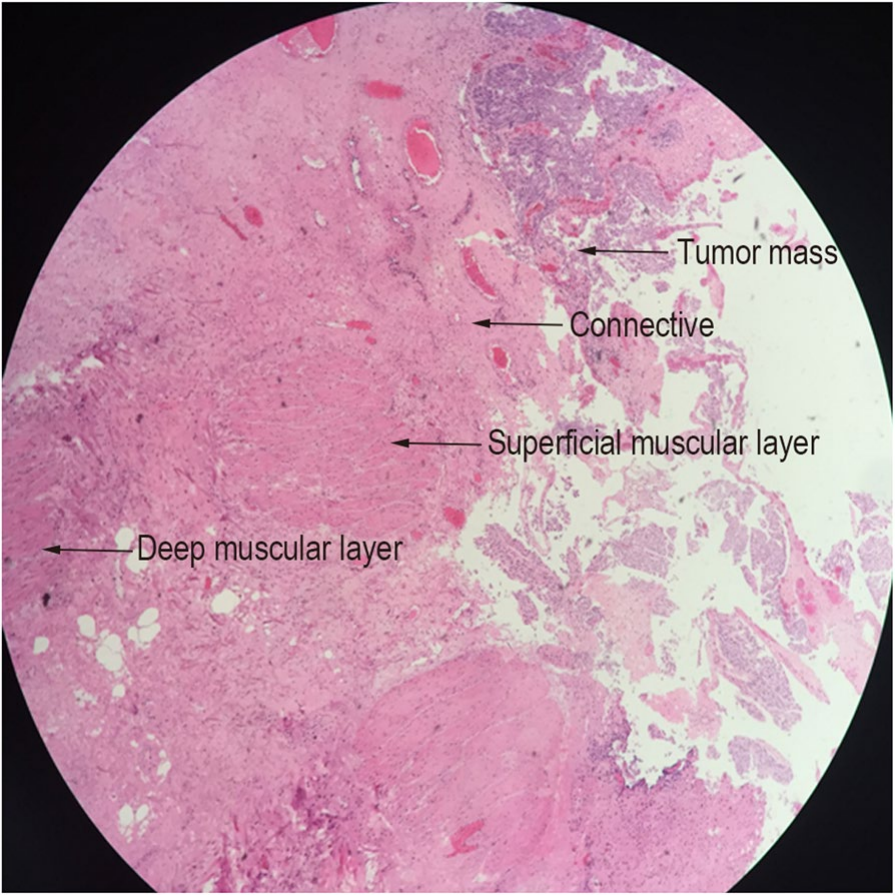
The H&E staining of a bladder tumor specimen from 980 nm diode laser en bloc resection. The superficial muscular layer and deep muscular layer are present

In this study, 88 patients with NMIBC underwent 980 nm diode laser en bloc resection of the bladder tumor. We found that the 980 nm diode laser could provide good hemostatic effects by means of a noncontact, point-to-surface pattern for obvious blood vessel bleeding. When the laser came into contact with the tissue, a precise cut with minimal bleeding was made. As a result, the bladder irrigation time was significantly shortened than that of PK-TURBT group, and none of the patients in the 980 nm laser group experienced delayed bleeding. The vaporization and cutting effects of the 980 nm diode laser were perfect. As shown in Fig. 1, the 980 nm diode laser controlled depth and width of cutting, as well as precise excision of the complete areas between the deep muscle layer and serosa layer. No complications of heat injury occurred. As previous literature described, the first step of laser treatment is making a circular incision in the mucosa around the tumor. However, we found that this step was difficult or unnecessary in bulky tumors or some special locations. In our opinion, the resection can start from the accessible tumor boundary, cut into the muscular layer, and then gradually expand in the areas between the muscular layer and the serosa. With the assistance of the sheath and water stream, we created an operative space between the tumor base and the serosa. The tumor was completely removed, thereby maintaining the integrity of the tumor. The bulky tumor could be easily removed from the bladder using the electrocautery resection loop. The accurate pathological results were obtained (Fig. 2). In this study, a total of 24 patients receiving PK-TURBT treatment were excluded because no muscularis tissue was found in the specimen. The 980 nm laser is safe to deal with the tumor in the lateral wall of the bladder for no obturator reflex occurring. In addition, there were some advantages compared with other lasers. The diode laser lacks a steam bubble effect similar to holmium and thulium lasers, which create steam bubbles whose energy may destroy tissue and affect visibility of operator [6]. And diode lasers have a smaller box size and a much higher wall-plug efficiency and a lower price [21].

There are some limitations in our study, including its retrospective nature and small sample size. And we investigated the patients treated in one hospital. The way in which the patients were enrolled may have introduced selection bias. Further prospective research needs to be carried out in the future.

## Conclusion

In conclusion, 980 nm diode laser is an efficient and safe tool in transurethral resection of NMIBC using the en bloc technique.

## Data Availability

All data generated or analyzed during this study are included in this published article.

## Abbreviations

TURBT: Transurethral resection of the bladder tumor
NMIBC: Non-muscle-invasive bladder cancer
TUR: Transurethral resection
ONR: Obturator nerve reflex
Ho:YAG: Holmium
Tm:YAG: Thulium
PK-TURBT: Plasmakinetic transurethral resection of bladder tumor
KTP: Potassium-titanyl-phosphate
TURP: Transurethral resection of the prostate
